# Interactions between Memory and New Learning: Insights from fMRI Multivoxel Pattern Analysis

**DOI:** 10.3389/fnsys.2016.00046

**Published:** 2016-05-26

**Authors:** Marlieke T. R. van Kesteren, Thackery I. Brown, Anthony D. Wagner

**Affiliations:** ^1^Department of Psychology, Stanford UniversityStanford, CA, USA; ^2^Section Educational Neuroscience, Faculty of Behavioural and Movement Sciences, Institute for Brain and Behaviour, Vrije Universiteit AmsterdamAmsterdam, Netherlands

**Keywords:** episodic memory, MVPA, Memory integration, fMRI, Memory encoding

## Introduction

Declarative memory—long-term memory for events and facts—is a key form of cognition that depends on distributed neural coding. Given the rich, multifaceted nature of life events, their neural representations (episodic memory “engrams”) typically incorporate a broad set of cortical and subcortical regions whose coding properties underlie event features (Paller and Wagner, 2002; Rugg et al., 2002; Tulving, 2002; Schacter et al., 2007). With continued experience, representations of individual events may form a foundation for more generalized semantic knowledge about the world (van Kesteren et al., 2012). A fundamental theoretical question is how existing memories interact with encoding of new experiences to enable formation of integrated knowledge structures.

The distributed nature of memory content in the brain, both locally (i.e., across neurons within a region) and across relevant cortical and subcortical regions, creates challenges for measurement of mnemonic content across various stages of memory encoding and retrieval. By combining non-invasive imaging techniques (e.g., functional magnetic resonance imaging–fMRI) with multivariate pattern-analyses (MVPA), such representational content can be decoded from distributed patterns of brain activity (Polyn et al., 2005; Norman et al., 2006; Rissman and Wagner, 2012). Moreover, quantitative measures of mnemonic representations can be related to behavioral performance measures, thus informing mechanistic models of memory.

At a macroscopic level, mnemonic representations of events are distributed across perceptual, motor, affective, and associative brain regions (Tulving and Markowitsch, 1997). Episodic memory retrieval entails the reinstatement or reconstruction of information encoded in memory (for reviews see Danker and Anderson, 2010; Ben-Yakov et al., 2015). MVPA provides a means of measuring distributed neural representations, and quantifying reinstatement processes (Norman et al., 2006; Rissman and Wagner, 2012). Importantly, a myriad of externally and internally generated retrieval cues can drive reinstatement of existing memory traces during encoding of related information. Such reinstatement may support the formation of more generalized knowledge through integration of new with old memories (Shohamy and Wagner, 2008; Preston and Eichenbaum, 2013). As such, elements of new memories that overlap with prior experiences can trigger reinstatement and integration processes allowing for extension and strengthening of existing associative knowledge structures, or “schemas” (Tse et al., 2007; van Kesteren et al., 2012).

The medial temporal lobe (MTL)—with the hippocampus at its core—is the most prominently studied region in memory research (Burgess et al., 2002; Squire et al., 2004; Eichenbaum et al., 2007). The hippocampus serves as an integrative hub for the binding of disparate neocortical representations of event features into unified memories (Eichenbaum et al., 2004; Andersen, 2007). Through creating flexibly addressable memory traces that link to the driving cortical representations of event content, the hippocampus can support subsequent reactivation of a remembered event's feature representations in the neocortex during retrieval. MVPA techniques can index expressions of distributed memory representations and processes in MTL as they unfold, as well as probe reinstatement and integration processes in content-selective cortical regions (Polyn et al., 2005; Johnson et al., 2009; Staresina et al., 2012; Gordon et al., 2014; Sigman et al., 2014).

Beyond the MTL, other cortical areas have been posited to contribute to across-event integration. In particular, the integration of associated memories is thought to also depend on computations within the medial prefrontal cortex (mPFC), a prefrontal region intimately connected with the hippocampus and suggested to be involved in the building of knowledge structures (van Kesteren et al., 2012; Preston and Eichenbaum, 2013). Recent evidence from direct neuronal recordings in non-human models of memory has linked hippocampus and mPFC population coding to the expression of schema knowledge (McKenzie and Eichenbaum, 2011; McKenzie et al., 2014; Richards et al., 2014). In humans, MVPA provides a powerful means to assess how mPFC and the hippocampus underlie integration of newly learned experiences with existing memories, and critically, to link this integration process with cortical reinstatement (Dudai and Eisenberg, 2004; Kuhl et al., 2010; Nadel et al., 2012).

Here we review how MVPA, applied to fMRI-data, is leveraged to address fundamental questions about reinstatement and subsequent integration of memory representations in the human brain. We discuss a framework in which reinstatement of prior knowledge during new learning can facilitate formation of integrated knowledge across experiences, highlight evidence for potentially disruptive effects of such processes on other expressions of memory (e.g., memory for episodic details), and suggest future research directions.

## Reinstatement as a mechanism for building integrated knowledge

Reinstatement of a previously learned memory during new encoding may build associations between overlapping experiences (Eichenbaum, 2000; Shohamy and Wagner, 2008; Kuhl et al., 2010; Preston and Eichenbaum, 2013; Schlichting and Preston, 2015), facilitating across-event generalization and construction of integrated knowledge structures (van Kesteren et al., 2012). Recent MVPA-studies have provided important insights into this phenomenon.

Univariate fMRI studies provided important initial evidence for integrative processing during associative learning within the hippocampus (Heckers et al., 2004; Shohamy and Wagner, 2008; Kuhl et al., 2010; Wimmer and Shohamy, 2012). Subsequently, MVPA studies demonstrated a relationship between mnemonic reinstatement during new learning and subsequent memory performance (Kuhl et al., 2011, 2012). Building on this literature, Zeithamova et al. (2012) used MVPA to directly examine how memory reinstatement in content-selective neocortical regions relates to behavioral integration measures. They found that the strength of cortical reinstatement of past events during new learning is related to behavioral expression of across-event integration. They further observed that hippocampal activity decreases as memories become integrated, while mPFC activity increases (Zeithamova et al., 2012). Interestingly, evidence for memory reinstatement in content-selective cortex during post-encoding periods has also been related to forming associations between events (Tambini et al., 2010), suggesting that off-line processing may also be important for mnemonic integration (Schlichting and Preston, 2014).

Progress in understanding the neural mechanisms governing integration has further come from MVPA studies examining how integration states relate to those of encoding, retrieval, and pattern separation. Integration putatively arises from a combination of encoding processes and reinstatement. However, recent evidence suggests that an “integrative state” may be dissociable from other mnemonic processes. In particular, the distributed neural patterns related to integration (a) are dissociable from those associated with separation processes in both the hippocampus and mPFC (Schlichting et al., 2015), as would be predicted given the contrasting function of separation in orthogonalizing (rather than integrating) memory traces; and (b) are also dissociable from singular encoding and retrieval states in an extended memory network including the hippocampus and mPFC (Richter et al., 2015). As such, integrated memories may be differentially represented than those learned in isolation.

## Reinstatement processes may impair episodic expressions of memory

While integration may enable generalization (Shohamy and Wagner, 2008; Schlichting and Preston, 2015) and sometimes further protect memories from forgetting (Kuhl et al., 2010; Schlichting et al., 2014), one potential negative consequence of integration is that memory for unique aspects of an event may suffer from greater interference. This could occur when integration of distinct representations results in a more generalized memory characterized by regularities across encoding events (van Kesteren et al., 2012; Sweegers et al., 2015). Reinstatement during encoding may also directly interfere with encoding details of new experiences (Kuhl et al., 2011), which could further favor the formation of generalized memories over ones rich in episodic detail. Indeed, integration can yield subsequent forgetting of episodic details, as is widely investigated in retrieval-induced forgetting and proactive and retroactive interference paradigms (Anderson et al., 1994; Levy and Anderson, 2002; Levy et al., 2010; Murayama et al., 2014). Furthermore, integration can lead to increased competition-driven retrieval failures (Smith et al., 1978; Wixted, 2004), and behavioral misattribution of stimuli from one experience to another (Hupbach et al., 2007; St. Jacques et al., 2013).

Recent MVPA fMRI studies have examined the relationship between reinstatement during encoding and interference during subsequent retrieval attempts, putatively leading to episodic detail loss. For example, researchers have shown that the degree to which prior memories are reinstated in content-selective cortex during new encoding predicts competition (Kuhl et al., 2011; Wimber et al., 2015) and misattribution (Gershman et al., 2013) between old and new memories. Additionally, while not directly a consequence of integration, Poppenk and Norman showed that brief reinstatement of a memory can lead to reduced similarity between its prior and subsequent neural representations (Poppenk and Norman, 2014); Prediction error signals may drive the mechanisms underlying such reinstatement-driven forgetting (Kim et al., 2014).

**Figure 1:**
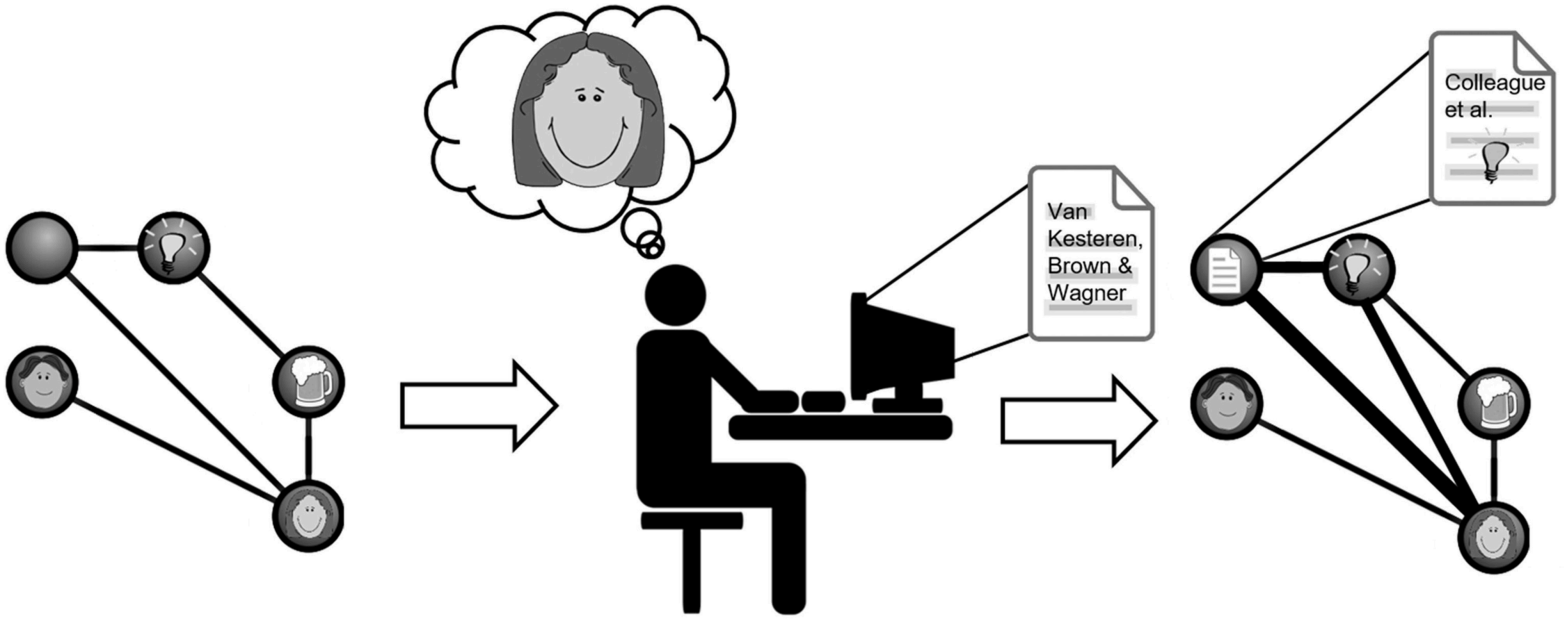
**Illustration of the potential generalizing and misattribution processes following reinstatement of prior knowledge during new learning**. Imagine you have a strong memory engram of a colleague and good friend of yours, which includes memories about her husband and discussing research ideas about reinstatement and memory over a beer. Then you read our current paper which makes you reinstate the memory of your colleague and her ideas. This can lead to a change in this existing engram, integrating the paper with it and generalizing its ideas to the ones you discussed in the pub. While this process may strengthen existing connections in the engram (depicted as thickening lines in the right engram representation), it can also increase the possibility of misattributing the author to be your colleague (depicted in the representation of the paper).

The above-discussed findings illustrate the complex nature of encoding-retrieval interactions during learning. From a theoretical perspective, more generalized “schematic” memories tend to lose episodic detail over the course of their formation. When considering the broader memory reactivation literature, one possibility is that interference effects contribute to such a loss of detail during both formation and retrieval of integrated memories. The data reviewed support a framework in which different facets of previously encountered or novel memories may be altered such that when distinct memory traces are integrated, associations representing detailed episodic experience-specific features might weaken or become distorted whilst those reflecting overlapping features may be strengthened (see Figure 1; Walker and Stickgold, 2010; Lewis and Durrant, 2011; Schlichting and Preston, 2015). In the context of integration, we propose that whether an individual memory is considered remembered or forgotten is in part a matter of how it is probed. Using MVPA to relate how memory representations change through integration and consolidation will yield important insights into how engrams are formed and the implications of integration for different memory expressions.

## Future directions

The results described above fit within a framework wherein our memory circuitry is drawn toward integrating new experiences with prior knowledge. This may change the earlier-acquired memory trace, perhaps (a) giving rise to between-trace interference or weakening of its associations, either or both of which would result in episodic details loss, but also (b) allowing for incorporating the new experience into a semantic memory engram (Dudai, 2012; Josselyn et al., 2015). With continued experience, such an engram (or “schema”) could acquire additional associations, further increasing its future probability of activation. New information that cannot be readily integrated with prior knowledge may be stored separately.

This perspective motivates new questions about the nature of mnemonic processing, questions that also can be answered using MVPA techniques. For example, instead of asking whether one memory is stronger or weaker than another, we can ask whether and how both new and existing memories change with integration, at both the neural and cognitive level: (a) Do they become more alike (Milivojevic et al., 2015) due to the merging of new information into a modified representation of existing memories, or (b) do existing memories keep their initially encoded blueprint, with an integrated representation forming as a novel memory trace that is distinct from the existing representation? In both cases, integrated memories may be represented through overlapping neural representations, possibly facilitating extraction and expression of across-memory regularities (Schapiro et al., 2012). What does this mean for the expression of memory details at retrieval? Given the importance of being able to distinguish between memories that share features with one another for decision-making (Brown and Stern, 2014), another critical question is how the brain balances integrating and disambiguating experience details. Answers to these questions can further inform understanding of the push-pull relationship between pattern separation and completion, as well as the episodic-to-semantic memory shift.

Better understanding of how memories build on each other to construct integrated or semantic memory engrams is of central importance in settings where they benefit our daily lives. For example in educational situations, where students are taught to construct knowledge through a specific training regimen, neuroscientific insights into the biological mechanisms underlying our learning abilities and interactions with prior knowledge are very valuable (Goswami, 2006; Howard-Jones, 2008; Sigman et al., 2014). Such insights may guide students and teachers to improve knowledge structure formation, while minimizing creation of misconceptions. This way, the neuroscience of memory may find its way into the classroom.

## Conclusion

The use of multivariate decoding and similarity techniques to inform memory research is rapidly advancing. Because of the distributed nature of episodic and semantic memory representations, MVPA-analyses are promising techniques for delineating how distinct mnemonic representations interact. Researchers have provided novel evidence for memory alterations that occur through mnemonic reinstatement during learning–changes that may serve to facilitate construction of integrated, generalizable knowledge about our world. However, when such integration occurs, it may come at the expense of episode-specific distinctions in memory. Insights from MVPA may help bridge perspectives on how knowledge structures form with constructs such as integrative encoding and mnemonic interference. Understanding neural and cognitive mechanisms contributing to memory integration may inform learning in real-world settings such as education, where efficient knowledge construction is imperative for success.

## Author contributions

All authors listed, have made substantial, direct and intellectual contribution to the work, and approved it for publication.

## Funding

This research is funded by a Rubicon fellowship from the Netherlands Organisation for Scientific Research and the Wallenberg Network Initiative on Culture, Brain, and Learning.

### Conflict of interest statement

The authors declare that the research was conducted in the absence of any commercial or financial relationships that could be construed as a potential conflict of interest.
